# Plasmonic Purcell effect reveals obliquely ordered phosphorescent emitters in Organic LEDs

**DOI:** 10.1038/s41598-017-01701-8

**Published:** 2017-05-12

**Authors:** R. Mac Ciarnain, D. Michaelis, T. Wehlus, A. F. Rausch, S. Wehrmeister, T. D. Schmidt, W. Brütting, N. Danz, A. Bräuer, A. Tünnermann

**Affiliations:** 1 Fraunhofer Institute for Applied Optics and Precision Engineering IOF, Albert-Einstein-Str. 7, 07743 Jena, Germany; 2 Institute of Applied Physics, Abbe Center of Photonics, Friedrich-Schiller-University Jena, Albert-Einstein-Str. 15, 07745 Jena, Germany; 3 OSRAM OLED GmbH, Wernerwerkstr. 2, 93049 Regensburg, Germany; 4 Institute of Physics, University of Augsburg, Universitätsstr. 1, 86135 Augsburg, Germany

## Abstract

The non-isotropic alignment of molecules can increase the interaction efficiency with propagating light fields. This applies to both emissive and absorptive systems and can be exploited for achieving unprecedented efficiencies of organic opto-electronic devices such as organic light-emitting diodes. Optical analysis has revealed certain phosphorescent emitters to align spontaneously in an advantageous orientation. Unfortunately, established approaches only determine an average orientation because emission patterns solely depend on the second moments of the transition dipole vector distribution. In order to resolve further details of such a distribution, additional differences in the emission characteristics of parallel and perpendicularly oriented emitters need to be introduced. A thin metal layer near the emitters introduces plasmon mediated losses mostly for perpendicular emitters. Then, analyzing the emission at different polarizations allows one to measure emission lifetimes of mostly parallel or mostly perpendicular oriented emitters. This should alter the transient emission when observing the temporal phosphorescence decay under different directions and/or polarizations. The angular width of the orientation distribution can be derived from the degree of such lifetime splitting. Our results suggest a narrow but obliquely oriented molecular ensemble of Ir(MDQ)_2_(acac) doped into the α-NPD host inside an Organic LED stack.

## Introduction

Starting from its first experimental observation^1^ the spontaneous alignment of phosphorescent emitters in organic light-emitting diodes (OLED) attracts continuous attention because of its strong effect on outcoupling efficiency^1–3^. Recently, such alignment has been observed for several emitters^4^ and was correlated with the permanent molecular dipole moments^4^, a strong formation of supra molecules due to an alignment of the triplet excited states within the host^5^, or the alignment of anisotropic molecules at the thin film surface during deposition^6^. Resulting efficiency enhancements have been reported for both phosphorescent guest-host systems^1, 2^ as well as emitters exhibiting delayed fluorescence^7–9^.

Different experimental approaches are conducted to analyze the emitter orientation distribution (EOD) of the emission transition dipole moments (TDM) in an OLED. Electroluminescence (EL)^1, 10^ or photoluminescence (PL)^1, 5, 6, 11^ emission patterns allow extracting the second moments of the EOD, provided that the experimental configuration allows one to observe sufficient emission from perpendicular emitters^12^. Alternatively, the analysis of the position dependent emission lifetime^13–16^ yields information about the EOD. This approach exploits the fact that the Purcell effect^17^ introduces orientation dependent emission rates, especially close to the metal cathode. However, such devices give very low intensity from perpendicularly oriented emitters. In this work we place an additional metal layer near the emission layer of an OLED to cause the lifetime splitting while retaining a microcavity, which enables high perpendicular emitter emission. This allows the separate observation of parallel and perpendicular emitter lifetimes via polarization filtering.

**Figure 1:**
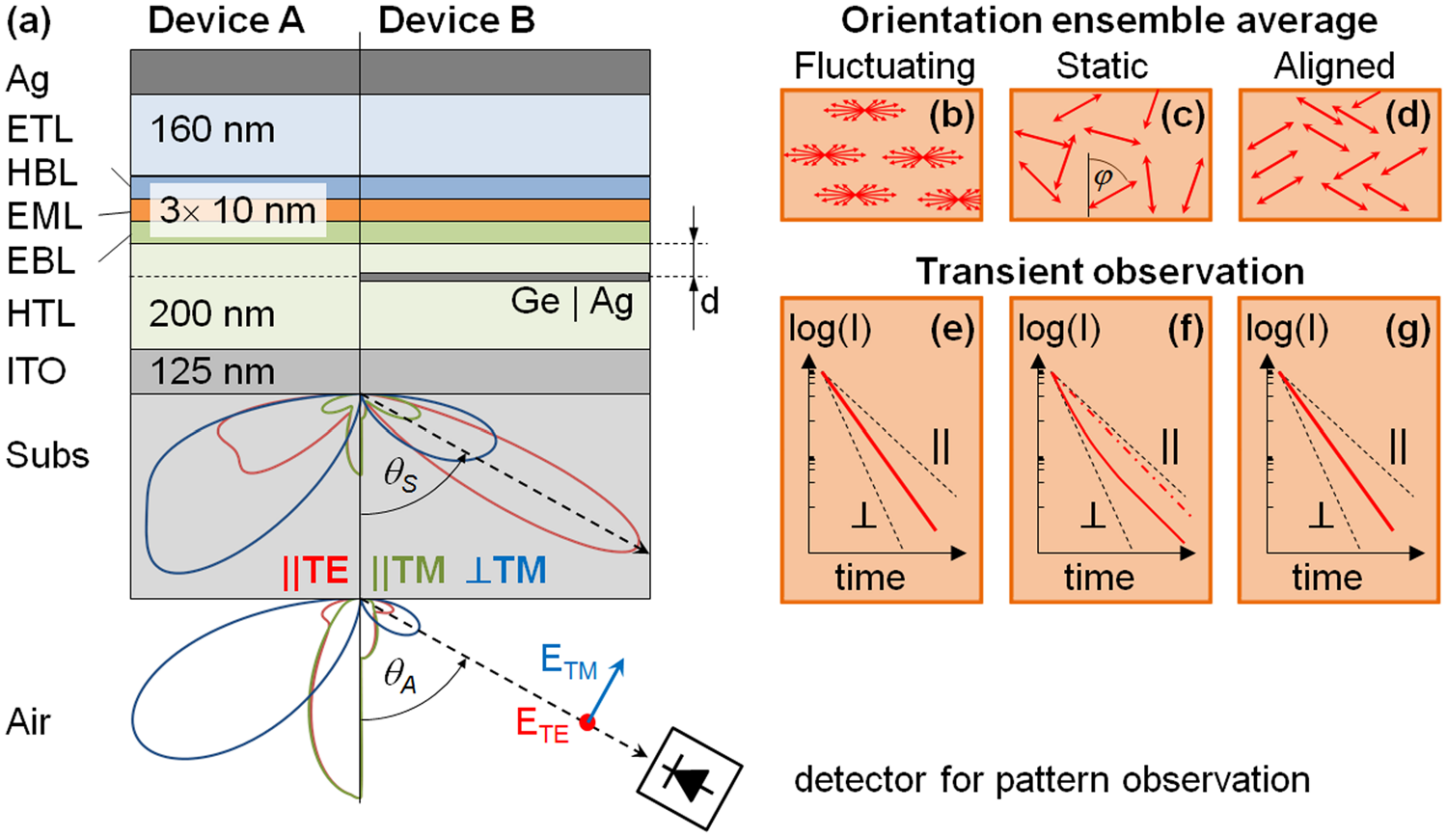
The geometry of the two OLED types (**a**) is shown with an illustration of the orientation averaging models (**b**,**c**,**d**) and the corresponding expected transient observation (**e**,**f**,**g**). In (**a**) the emission patterns generated by the three orthogonal dipoles are plotted for the d = 30 nm case inside the substrate (angle θ_s_ with respect to the normal, accessed experimentally by half ball lens coupling) or in air (angle θ_A_). Observation direction and orientation of the polarized electric field are shown for convenience. The expected temporal evolution of the intensity observed in a transient experiment is sketched for the fluctuating (**b**), static (**c**) and aligned (**d**) cases in diagrams (**e**), (**f**) and (**g**), respectively. The latter diagrams plot the transient intensity observed for strictly parallel (||) or perpendicular (⊥) emitters (black dashed) along with the TE and TM experimental observations (straight red). Diagrams (**e**,**g**) illustrate that TE and TM measured lifetimes for these cases are expected to be the same within our resolution, while diagram (**f**) additionally includes a second experimental time trace (dash dot) expected for a different observation direction.

In order to extract more details of the EOD of the TDMs this measurement was combined with a standard emission pattern analysis. The combination of the heteroleptic red phosphor Iridium(III)bis(2-methyldibenzo-[f,h]quinoxaline)(acetylacetonate) [Ir(MDQ)_2_(acac)] emitter doped into an N,N0-bis(naphthalen-1-yl)-N,N0-bis(phenyl)-benzidine [α-NPD] host matrix, which has previously been found to exhibit spontaneous alignment of the emitters^1, 13^, is analyzed in a device geometry according to Fig. 1. A well-selected thickness of the electron transport layer (ETL) ensures significant emission from perpendicularly aligned emitters^12^, thus enabling the quantification of the second moments of the EOD (see Device A in Fig. 1a). An additional semi-transparent plasmon-supporting thin metal (PSTM) layer on the anode side of the emitter introduces large lifetime differences between parallel and perpendicularly aligned emitters (see Device B in Fig. 1a). This causes plasmon-mediated losses especially for perpendicular emitters^18^, resulting in a reduced lifetime compared to parallel emitters due to orientation dependent Purcell factors.

Any transient experiment will yield temporal decays of the spontaneously emitted intensity with emission lifetimes in-between those of strictly parallel or perpendicular emitters (red curves in Fig. 1e,f,g). The detailed temporal behavior depends on the EOD of the TDMs combined with a weighting function, which considers the contribution of each emitter TDM to the experimental result. The emission patterns in Fig. 1a illustrate the effect of the observation conditions on the detection efficiency for the three basic orthogonal TDM directions. Observation at approximately 60° in the substrate of Device B with PSTM layer will allow us to observe mostly parallel emitters with transverse electric (TE, red pattern in Fig. 1a) and mostly perpendicular emitters with transverse magnetic (TM) polarization. In the latter case, a superposition of parallel and perpendicular contributions will be present (green and blue pattern in Fig. 1a). Therefore, one can combine the orientation selectivity of the emission pattern and the emission lifetime in order to derive additional details on the TDM alignment of the emitting ensemble.

In this paper, the theory to describe such an experimental approach will be discussed first, taking the nature of different emitters into account. Then, established emission pattern analysis will be used to verify the emitter properties prior to performing the emission lifetime analysis for different observation conditions. This utilizes the plasmonic Purcell effect in a specialized micro cavity for EOD analysis. Subsequently, the experimental results will be discussed in order to derive conclusions for the EOD of the emitter ensemble under study.

## Theory and Stack Design

**Figure 2:**
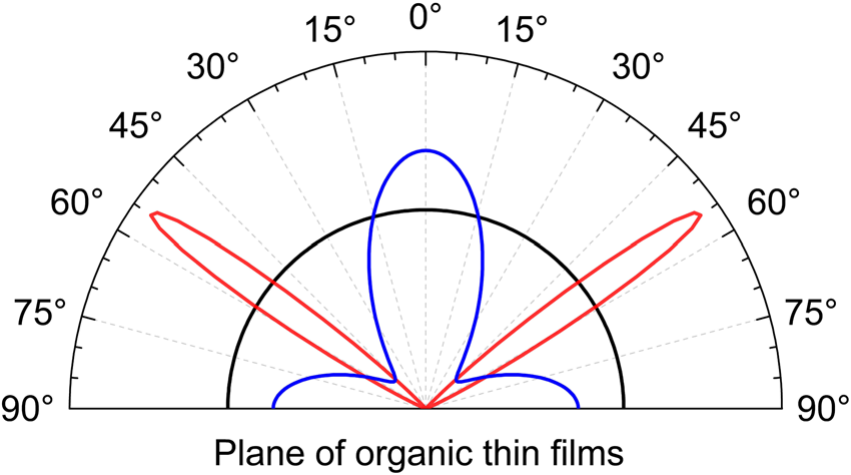
Three non-normalized examples of orientation distributions exhibiting the orientation ratio ${\langle {p}_{||}^{2}\rangle }_{\phi }:\,{\langle {p}_{\perp }^{2}\rangle }_{\phi }=2\,:\,1$ of an isotropic distribution (black). The two additional curves illustrate a well-aligned ensemble (red) and an ensemble consisting of two Gaussian shaped parallel and perpendicular components (blue).

The geometry of the OLED stack is assumed to be isotropic in the plane of the layers, i.e., to exhibit a uniaxial symmetry. Then according to electromagnetic theory^12, 19^ the classical, stationary emission pattern of an emitting dipole with fixed, normalized parallel *p*_‖_(*φ*) = sin(*φ*) and perpendicular ${p}_{\perp }(\phi )=\,\cos (\phi )$ TDM components exhibiting the angle φ with respect to the normal of the stack interfaces is given by

1 $${I}_{class}(\theta ,\phi )=[{I}_{TE}^{||}(\theta )+{I}_{TM}^{||}(\theta )]\cdot {p}_{||}^{2}(\phi )+{I}_{TM}^{\perp }(\theta )\cdot {p}_{\perp }^{2}(\phi ),$$

where ${I}_{TE,TM}^{||,\perp }$ are the intensities of horizontally (||, φ = π/2) or vertically (⊥, φ = 0) aligned and continuously oscillating dipoles observed in the far field under the given polarization (TE, TM) and detection angle *θ* (see Fig. 1a). The intensities ${I}_{TE,TM}^{||,\perp }$ contain the stack interference properties as well as the typical dipole radiation characteristics that we will exploit. Please note that the value of θ is different in air or substrate due to refraction at the glass-air interface. According to Eq. (1) oblique emitters in a real ensemble can be always decomposed into a perpendicular and a parallel dipole contribution, where all classical characteristics depend linearly on the square of the corresponding TDM components. Thus, an ensemble averaging ${\langle Y\rangle }_{\phi }=\int Y(\phi )\cdot f(\phi )\cdot \,\sin \,\phi \cdot d\phi$ of any classical power quantity *Y* with respect to the emitter orientation angle *φ* and the angular EOD *f(φ)* will depend linearly on the second moments of the TDM components

2 $$\begin{array}{rcl}{\langle {p}_{||}^{2}\rangle }_{\phi } & = & \int {p}_{||}^{2}(\phi )\cdot f(\phi )\cdot \,\sin \,\phi \cdot d\phi \\ {\langle {p}_{\perp }^{2}\rangle }_{\phi } & = & \int {p}_{\perp }^{2}(\phi )\cdot f(\phi )\cdot \,\sin \,\phi \cdot d\phi \end{array}$$

only. The example of an isotropically distributed ensemble exhibits the EOD $f(\phi )=1$ that is associated with the orientation ratio, i.e., the ratio of parallel to perpendicular dipole contributions, of ${\langle {p}_{||}^{2}\rangle }_{\phi }:\,{\langle {p}_{\perp }^{2}\rangle }_{\phi }=2\,:\,1$. Figure 2 illustrates this case along with two non-isotropic EODs exhibiting the same orientation ratio. This example illustrates the limited information that can be gained from classical orientation analyses^1, 5, 6, 10, 11^, and points out the need to improve the characterization method in order to differentiate such qualitatively different distributions.

Extending the characterization to emission lifetime based analyses can add qualitatively new features and thus improve the characterization method. Similar to the intensity in Eq. (1) the orientation dependent decay rate Γ (the inverse of emission lifetime *τ* = 1/Γ) of a single excited state depends on the rates of the strictly parallel (||) and perpendicular (⊥) emitters according to

3 $$\frac{{\rm{\Gamma }}(\phi ,q)}{{{\rm{\Gamma }}}_{0}}=\frac{{{\rm{\Gamma }}}_{||}(q)}{{{\rm{\Gamma }}}_{0}}\cdot {p}_{||}^{2}(\phi )+\frac{{{\rm{\Gamma }}}_{\perp }(q)}{{{\rm{\Gamma }}}_{0}}\cdot {p}_{\perp }^{2}(\phi )$$

**Figure 3:**
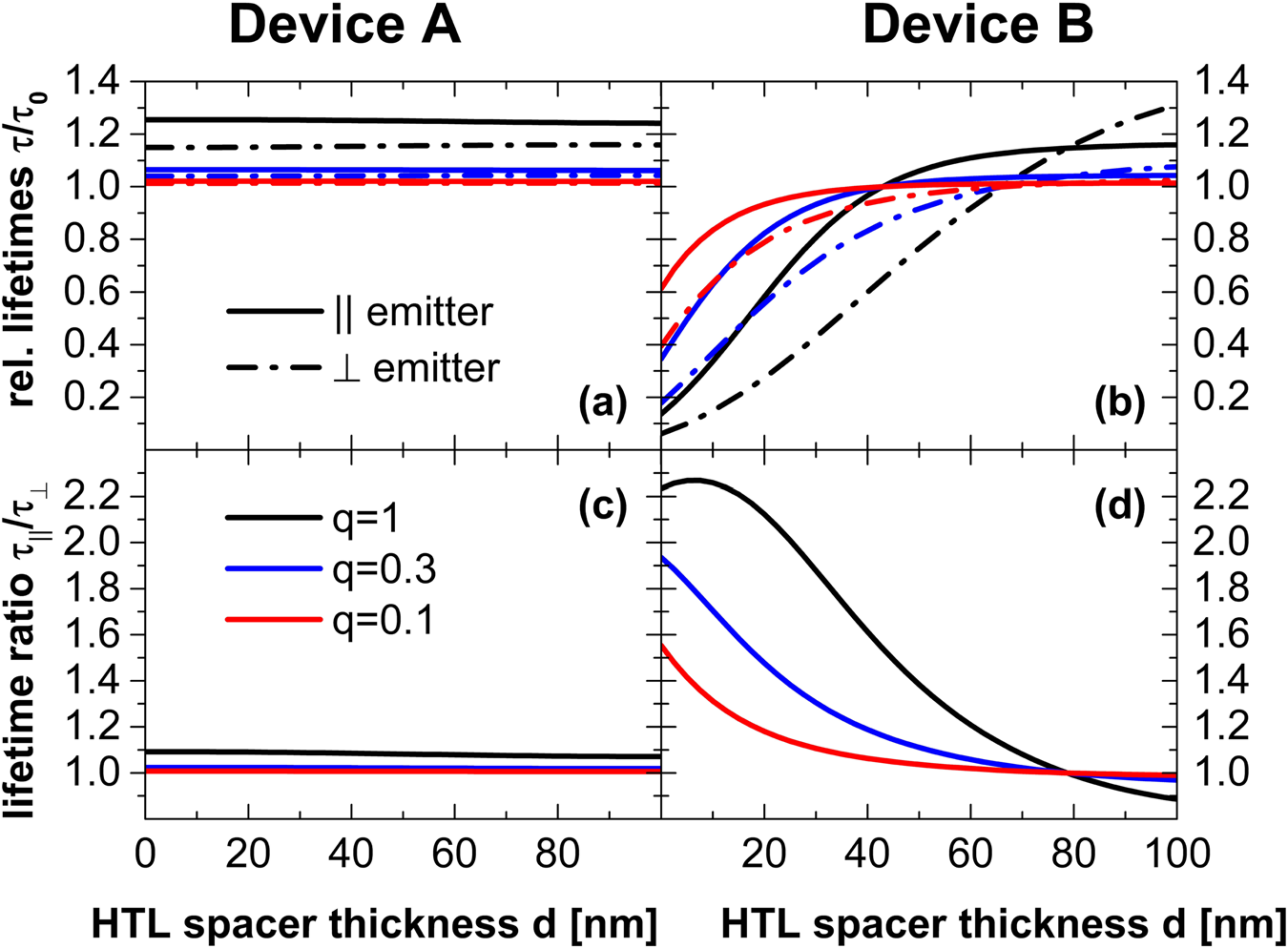
The simulated emission lifetimes for parallel (||, straight) or perpendicularly (⊥, dash dot) aligned emitters in devices A (**a**) and devices B (**b**) are plotted vs. HTL spacer thickness d for the cases q = 1 (black), q = 0.3 (blue) and q = 0.1 (red). The ratio of the lifetimes is calculated from these data and is shown in diagrams (**c**, device A) and (**d**, device B). Devices with *d*=10, 30, and 75 nm are analyzed experimentally.

It is determined relative to the emission rate Γ_0_ in an infinitely extended, isotropic emissive medium. For various typical OLED configurations the decay rate of parallel and perpendicular emitters are similar ${{\rm{\Gamma }}}_{\parallel }\cong {{\rm{\Gamma }}}_{\perp }$ and thus, any transient behavior contains only minor information about dipole orientation (Fig. 3a,c). But exploiting the Purcell effect, especially due to strong plasmonic absorption of emitters close to an PSTM layer, large decay differences can be introduced ${{\rm{\Gamma }}}_{\parallel }(q)\ll {{\rm{\Gamma }}}_{\perp }(q)$. Figure 3(b,d) illustrates that the amount of plasmonic absorption strongly scales with the distance between the PSTM layer and the emitter. Additionally, the emission rates are affected by the emitter’s quantum efficiency *q* when taking non-radiative depopulation of the excited state into account^13, 14^. Figure 3(d) illustrates that reduced emitter quantum efficiency decreases the lifetime splitting because the non-radiative decays will progressively prevail against the radiative ones, thus reducing the differences between parallel and perpendicular decay rates.

Combining equations (1) and (3) yields the temporally resolved intensity *I*_*S*_ of an emitting molecular ensemble according to

4 $${I}_{S}(\theta ,t,q) \sim {\langle \frac{{I}_{class}(\theta ,\phi )}{{\rm{\Gamma }}(\phi ,q)}\cdot \exp \{-{\rm{\Gamma }}(\phi ,q)\cdot t\}\rangle }_{\phi }.$$

This description assumes each molecule to exhibit a unique decay rate ${\rm{\Gamma }}(\phi ,q)$ that depends on its individual, fixed angle of orientation φ and will be referred to as “static” (subscript S) ensemble average (Fig. 1c). In cases with ${{\rm{\Gamma }}}_{||}\ne {{\rm{\Gamma }}}_{\perp }$ the TDM components enter Eq. (4) none-trivially, resulting in non-exponential time traces (Fig. 1f). Details about the shape of the distribution function *f*(*φ*) can be obtained from such experimental data. A mono exponential temporal behavior is observed only in the very special situations of either strictly aligned molecules (Fig. 1d,g) or orientation independent emission rates ${{\rm{\Gamma }}}_{||}={{\rm{\Gamma }}}_{\perp }$. Such a static ensemble average has been observed in photo luminescing polymer films doped with Rhodamine 6G, which is assumed to have a single emission TDM and has an emission lifetime of approximately 3 ns^20–22^. It yields, in general, non-exponential time traces (Fig. 1f) of emitting ensembles^23^.

Contrarily, Europium complexes of 1 ms emission lifetime are well known not to exhibit orientation dependent emission lifetimes in the vicinity of metal mirrors^24^. The average decay rate observed experimentally can be described by the “fluctuating” (subscript F) ensemble average

5 $${I}_{F}(\theta ,t) \sim \frac{{\langle {I}_{class}(\theta ,\phi )\rangle }_{\phi }}{{\langle {\rm{\Gamma }}(\phi ,q)\rangle }_{\phi }}\cdot \exp \{-{\langle {\rm{\Gamma }}(\phi ,q)\rangle }_{\phi }\cdot t\}.$$

This observation can be associated with a thermal motion of the emitting ion during the emission lifetime. It can only yield a mono exponential temporal decay such as illustrated in Fig. 1(b,e). With regard to electroluminescent phosphors, such a fluctuating average has been reported for single homoleptic Iridium complexes^25^ due to the fact that degenerate TDMs can be assigned to each one of the three ligands. So depending on the nature of the emitting species and the orientation ensemble average, transient experiments will lead to different interpretations.

In order to experimentally visualize lifetime differences within the emitter ensemble two requirements need to be met: (i) Emission from perpendicular TDM components needs to be sufficiently visible, which is achieved by properly adjusting the emitter–cathode distance according to the design rules for EOD measurement^12^. (ii) The difference in the emission rates of parallel (Γ_||_) and perpendicular (Γ_⊥_) dipoles according to Eq. (3) needs to be as large as possible in order to be able to observe the desired effect.

Both requirements can be met in a state-of-the-art OLED stack as shown in Fig. 1. It comprises a glass substrate with an ITO anode that is coated sequentially with an organic stack consisting of hole transport (HTL), electron blocking (EBL), emissive (EML), hole blocking (HBL), and electron transport (ETL) layers. Finally, the stack is covered with an opaque Silver (Ag) cathode and encapsulated. An ETL thickness of 160 nm ensures the visibility of perpendicular emitters to meet condition (i). Usually, such a stack features emission rates that are almost independent of emitter orientation, ${{\rm{\Gamma }}}_{||}\approx {{\rm{\Gamma }}}_{\perp }$ (see Fig. 3). Thus, in order to meet requirement (ii), a Ge:Ag PSTM layer is introduced at different distances from the EML. This layer creates large plasmon mediated losses in the vicinity of the emitter, primarily for perpendicular emitters. Figure 3 illustrates that the relative emission lifetime is nearly independent of emitter orientation for all non-PSTM devices A and for PSTM devices B with d = 75 nm, whilst the other PSTM devices B with 10 nm and 30 nm thick HTL spacer layers should introduce significant emission lifetime splitting of up to a factor of two.

## Methods

### OLED preparation

Six OLED samples have been prepared by sequentially evaporating organic layers onto commercial ITO coated substrates under high vacuum conditions as described previously^1^. After depositing a 200 nm thick HTL, half of the samples were coated with a 11 nm thick Ag layer. The formation of Ag islands has been reduced by depositing a 1 nm thick Ge layer^26^ prior to Ag deposition. So devices with and without a PSTM layer can be compared directly. Then, all samples were coated with a further HTL as a spacer layer to the emitters (comprising thicknesses of d = 10 nm, 30 nm, or 75 nm) prior to depositing the emitting system (EBL/EML/HBL, each 10 nm thick) which is then covered with the ETL and cathode (Fig. 1). Regarding previous experiments^1^, the present devices differ in that HTL doping concentration has been increased to permit device operation with the PSTM layer inside the HTL, emitter concentration has been reduced to 3% (w/w), and the device active area has been increased to 1.68 cm².

### Stack analysis

Refractive index dispersion has been determined on single supported thin films^27^. Layer thicknesses have been extracted from spectral reflectivity measurements performed after each deposition step as well as with the complete devices. This revealed that the optical properties of the thin Ag layer are not consistent with published optical constants^28^, and that thickness dependent effects in the dispersion need to be considered^29^. Furthermore, the presence of Germanium introduces significant modifications of the dispersion as pointed out in ref. 30. Thus, a modified dispersion of the thin Ag layer is assumed for the simulations. The dispersion data of the thin Ag layer is available online in Supplementary Figure S1 along with reflectivity data of the stacks in Supplementary Figure S2.

### Electrodynamic simulations

All simulations and data fitting, both for stack analysis and luminescence modelling, have been performed by means of a self-written tool based on the dyadic Green’s functions approach as applied for previous studies^1, 12, 20, 31^.

### EL experiments

Continuous EL excitation was achieved by a constant current source (GS610, Yokogawa) generating a current density of 5 mA/cm^2^ in the device.

### PL experiments

Excitation utilized a 515 nm diode laser in order to excite the dye directly without pumping the matrix material, which was equipped with a linear polarizer and a half wave plate. The latter allows one to adjust polarization without introducing large intensity changes when switching polarization. The laser source was modulated with square pulses of 100 µs duration and 5 kHz repetition, resulting in 30 mW cw equivalent power.

### Luminescence detection

In order to achieve angularly resolved analysis the OLED sample was mounted on a rotational stage (Thorlabs). Luminescence emission spectra were recorded using a wire grid linear polarizer (NT47-101, Edmund Optics) with attached achromatic wave plate (AQWP05M-630, Thorlabs) and combined with a fiber coupled calibrated spectrometer (SD2000, Ocean Optics). Transient luminescence time traces were recorded by means of a photomultiplier tube (Hamamatsu R6356, 2 ns response) and an oscilloscope (LeCroy WR 6051 A, 2 ns resolution). A long pass filter (Schott OG 590) was used to block scattered laser light in PL excitation.

## Results

### Emission pattern analysis

**Figure 4:**
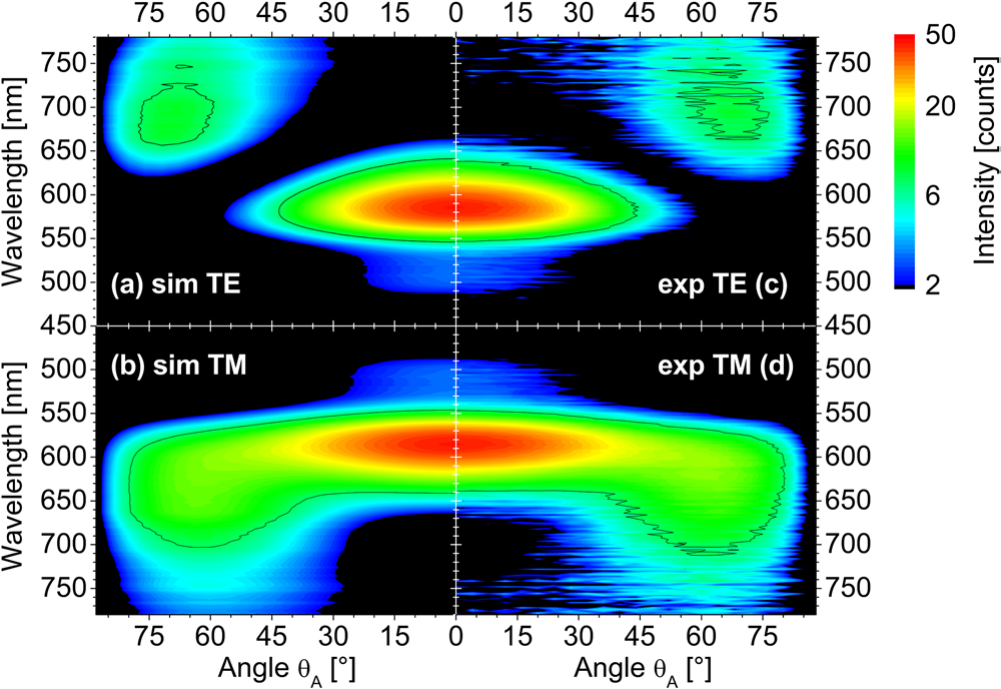
Polarized emission patterns are shown in logarithmic false color representation vs. observation angle in air θ_A_ and wavelength for device B with d = 30 nm. The four diagrams compare TE (**a**,**c**) and TM polarized (**b**,**d**) experiments (**c**,**d**) with simulation (**a**,**b**). A black isoline is shown for convenience. The increased experimental noise level is related to the reduced emission in air, as predicted in the emission patterns shown in Fig. 1(a).

Different experimental approaches have been combined to obtain a comprehensive model of the emission patterns and the transient luminescence behavior. First of all, the steady-state electroluminescence emission patterns have been analyzed to extract the emitter orientation ratio in the established way^1^. Figure 4 illustrates the resulting experimental and simulated emission patterns on a logarithmic scale for one Device B after such analysis. Due to the presence of the PSTM layer, the far field intensities observed in air are small, as also predicted in Fig. 1(a). However, we wish to point out that both kinds of devices yield a similar orientation ratio of ${\langle {p}_{||}^{2}\rangle}_{\phi }:{\langle {p}_{\perp }^{2}\rangle}_{\phi }=2:0.69$, which is well consistent with previously published values^1, 4^. Cross section data at a wavelength of 650 nm, i.e. intensity vs. observation angle, are available online in Supplementary Figure S3 for convenience. Thus, the samples exhibit the expected aligned emitter ensemble and no effect of the PSTM layer on the orientation ratio is observed.

### Transient lifetime analysis

According to previous findings^1^ no effect of the pump polarization on the emitted intensities has been found. This means that even if 0° TE excitation is applied, emission from all emitter orientations occurs as if these were uniformly excited. This observation is attributed to an orientational redistribution of the excitation energy due to internal molecular relaxation after excitation.

**Figure 5:**
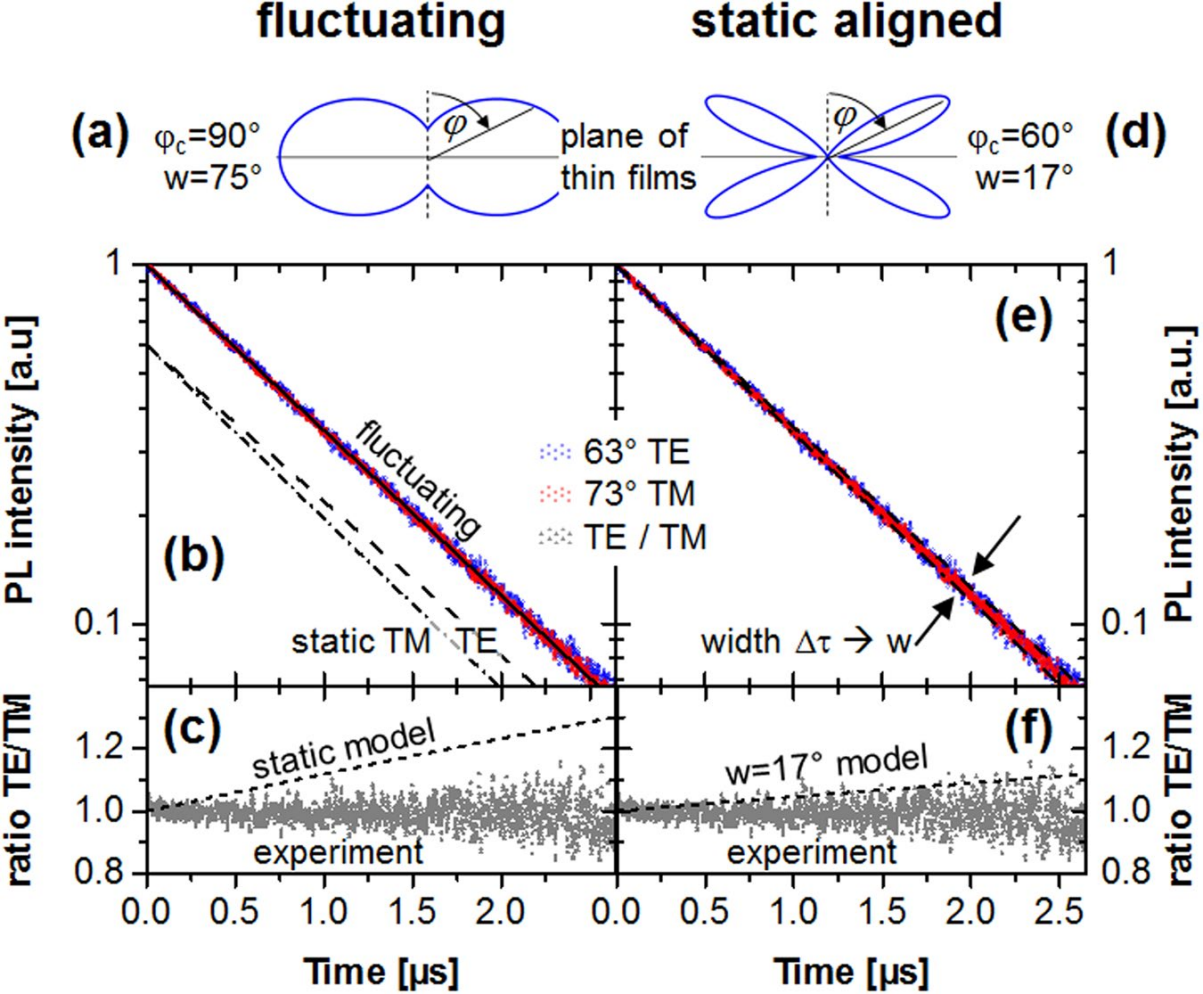
Analysis of one experimental data set obtained for device B with d = 30 nm to analyze the EOD of the emitter ensemble. Dots represent experimental data for TE θ_S_ = 63° (red), TM θ_S_ = 73° (blue) as well as their ratio (grey). The fluctuating average (**a**) is modelled by a horizontally centered, wide Gaussian function and predicts a single exponential decay (**b**), the decay time of which (straight black line, τ = 0.94 µs) would change in case of a static ensemble average to TE (dashed, τ = 0.89 µs) and TM (dash dot, τ = 1.00 µs). The same fact is well illustrated by the ratio of TE and TM polarized intensities in (**c**). Alternatively, assuming an aligned ensemble with a narrow Gaussian distribution (**d**) models the experimental data well (**e**,**f**). From the experimental errors an upper limit of the distribution width of about 17° can be deduced. Figures (**a**,**d**) illustrate the distribution function assumed in the model.

Transient data of one temporally resolved PL experiment is shown in Fig. 5, further data sets are available online in the Supplementary Figure S4. Different observation conditions have been used to preferably detect emitters with either parallel or mostly perpendicularly aligned transition dipole moments. However, both transient data sets are indistinguishable. Irrespective of uncertainties of the fit, this fact is well visualized by the temporally constant ratio of the experimental data (grey dots in Fig. 5c,f). Thus, no observation dependent lifetime was found.

**Figure 6:**
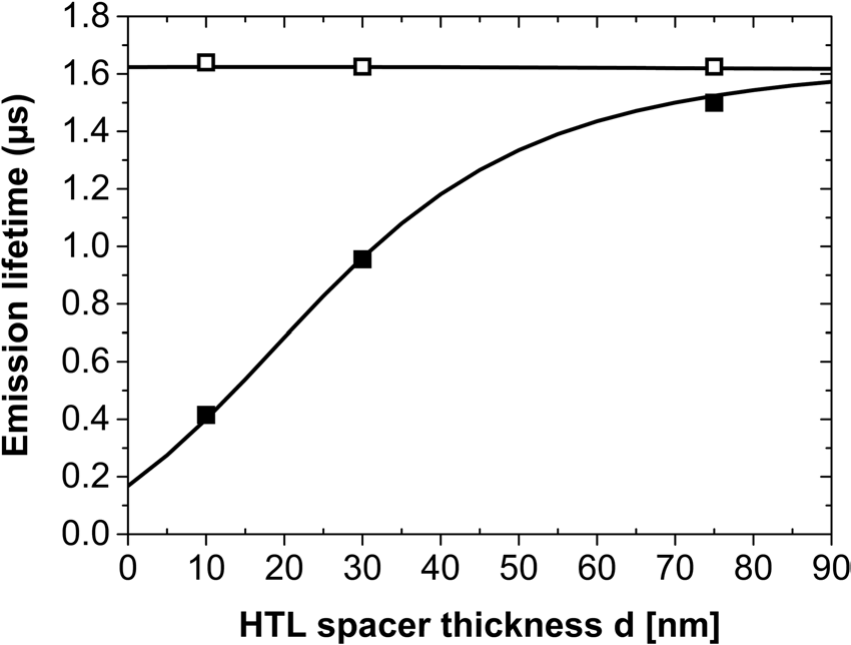
Emission lifetimes of Devices A (empty symbols) and B (filled symbols) plotted vs. HTL spacer layer thickness d. The simulated curves illustrate the best fit according to Eq. (5) with q = 0.83 and 1/Γ_0_ = 1.37 µs. Symbol size is set as a rough guide to experimental errors of Δd < ±3 nm and Δτ < ±0.02 µs. Note that no dependence of lifetime results on the polarization has been observed within the experimental accuracy.

Emission decay lifetimes were measured on samples of type A and B with three different spacer thickness. The results permit one to extract both the intrinsic quantum efficiency q and the emission lifetime in the infinite homogeneous medium τ_0_ = 1/Γ_0_, by fitting the data with Eq. (5). The fit in Fig. 6 matches the experimental data almost perfectly and reveals an intrinsic quantum efficiency of 83% along with 1/Γ_0,PL_ = 1.37 µs. Both, the high PL intrinsic quantum efficiency and emission lifetime agree well with previous studies^13, 31, 38^. According to the theory (Fig. 3) a minimal effect of HTL layer thickness on the lifetime is apparent for non-PSTM layer devices A, whilst the specialized micro cavities (devices B) feature a pronounced lifetime decrease with decreasing spacer thickness d.

## Discussion

The EL emission pattern based orientation analysis (Fig. 4b,d and Supplementary Figure S3) yields the expected preferred parallel EOD of the emitting TDMs. As outlined in the introduction, only the relative emitter contributions of parallel or perpendicular alignment can be extracted. So any EOD model is an assumption and cannot be further quantified by this experiment. Therefore, one could exemplarily assume two different situations to model the experimental emission patterns: (i) A Gaussian EOD

6 $$f(\varphi )\cong \exp \{-{(\varphi -{\varphi }_{c})}^{2}/{w}^{2}\}$$

centered in the plane of layers *φ*_*c*_ = 90° and exhibiting an approximate width *w*~75° (Fig. 5a). This corresponds to an “almost isotropic” ensemble with slightly less perpendicularly aligned emitters. (ii) Another valid assumption would be to align all molecular TDMs at an “average” angle $\bar{\phi }\approx 59.6^\circ$, which corresponds to a perfectly aligned ensemble. Note that the latter value is only slightly above the one of ${\bar{\phi }}_{iso}\approx 54.7^\circ$ obtained in fluorescence anisotropy measurements of isotropic ensembles. With regard to the emission pattern analysis performed for devices A both assumptions yield the same fit to the experimental data.

Next, emission lifetime analysis can be used for judging these different assumptions. The large emitter quantum efficiency obtained in the PL case yields a TDM orientation lifetime splitting by a factor two (compare Fig. 3). However, all experimental observations yield a single exponential time trace that contradicts the presence of multiple, sufficiently different single exponential decays associated with different orientations. Therefore a fluctuating ensemble average exhibiting the emission rate ${\langle {\rm{\Gamma }}\rangle }_{\phi }$ according to Eq. (5) might be deduced, which fits the simulations shown in Fig. 5(b,c) well. Previous single molecule experiments^25^ as well as x-ray scattering based analyses of molecular orientation^33^ of homoleptic emitters could support such a conclusion. But computational models of the TDMs in the heteroleptic emitter Ir(MDQ)_2_(acac) show, in contrast to the homoleptic Ir(ppy)_3_ molecule, overlapping TDMs of the two ligands^5^. This means that each molecule possesses practically only one possible TDM direction, thus ruling out a fluctuating ensemble average.

Intermolecular transfer processes could also cause orientation randomization. But transfer of the excitation energy to the EML matrix or neighboring layers are not expected because of the increased energy gap of these materials, which is experimentally apparent by missing absorption at wavelengths equal or larger than the excitation. Interactions of excited emitter molecules are mediated by Förster transfer, the radius of which has been reported to be in the 2 nm range^34^. Such a value is approximately a factor two below the average emitter separation in the present study. Furthermore, concentration dependent effects should be apparent in case of excited state interactions. These have been observed in a different host for emitter molecule concentrations higher than 18%^35^, which is well above the 3% dye doping used here.

According to these arguments, the static ensemble average applies. Then, the single exponential emission lifetime observed indicates a rather narrow distribution of phosphorescence emission lifetimes. As different observation conditions yield the same emission lifetime, a narrow oblique angular distribution of the TDMs is deduced. The assumption shown in Fig. 5(d) illustrates such a case when supposing the EOD to be of a Gaussian shape. Increasing the width of such a narrow EOD will increase the differences observed in the emission lifetimes outside of our experimental error. Therefore, from the uncertainty of the emission lifetime measurement (Δτ ± 0.02 µs) a reasonable limit for the EOD width $w\le 17^\circ$ can be extracted. The corresponding simulations shown in Fig. 5(e,f) suggest a rather well oriented TDM ensemble for the emitter under study.

The x-ray approach^33^, which is expected to be applied to further molecular systems, might be one tool to independently cross check our findings. Alternatively, electron spin resonance spectroscopy (ESR) using transition metal ions as probes due to their paramagnetic properties has been applied previously to indirectly measure molecular orientation distribution. An EOD width of w = 30°, with $\overline{{\rm{\phi }}}$ = 80°, was reported for a Langmuir Blodgett film doped with phthalocyanine^36^. Similar experiments on CuPcX4^37^ resulted in a much narrower EOD width of w = 5°, with $\overline{{\rm{\phi }}}$ = 80°. Azumi *et al*.^38^ measured a similar system to have EOD width w ≈ 14°. This comparison with highly ordered Langmuir Blodgett films illustrates that the degree of emitter orientation, which would be deduced when assuming the static orientation average, is unexpectedly high. So the findings should be checked independently by additional experiments such as x-ray scattering or ESR.

## Conclusions

The interpretation of OLED EOD measurements depends on the averaging of all the orientations in the real emitter ensemble. Such effects will become more pronounced when introducing lateral structures in an OLED, e.g. in the case of grating or scattering based outcoupling enhancement. Here, OLEDs featuring spontaneously aligning, heteroleptic Ir(MDQ)_2_(acac) phosphors have been analyzed. The device geometry is chosen to give sufficient emission from perpendicular emitters. The parallel vs. perpendicular emitter contribution of the ensemble has been detected by means of the established emission pattern method, in agreement with previous results. Additionally, a thin metal layer has been introduced in the vicinity of the emitter. This causes a pronounced increase of the decay rate of perpendicularly oriented emitters due to energy transfer into surface plasmons at this metal layer. When observing mostly parallel or perpendicular emitters at different angles and polarizations, no emission lifetime difference has been found despite the presence of such an orientation selective quenching effect.

This observation can be attributed to either a wide EOD (Fig. 5a) featuring a fluctuating ensemble average, or a well oriented narrow EOD (Fig. 5d) exhibiting a static orientation ensemble average. Due to the fact that the TDMs of the excited ^3^MLCT (metal-to-ligand charge-transfer) complex practically overlap to give one possible direction, we conclude a well aligned, narrow EOD of the phosphors in the EML. Experimental errors of our emission lifetime measurement indicate the upper limit of such a normal distribution width to be ±17°. As this value is unexpectedly low for a thermally evaporated guest-host system, more efforts should be undertaken to clarify the orientational behavior of the emitter luminescence. With regard to molecular orientation ESR spectroscopy or x-ray analysis could be used for verification.

The present work suggests a route to obtain more information about the photo-physical processes and the emitter properties from optical analyses of OLED patterns. Especially the EOD, which promises a potential of up to 50% external device efficiency increase in the OLED case, is a key parameter for future optimization of all related organic electronic technologies and applications. Currently, orientation analysis and optimization are based on measuring the ratio of the EOD’s second moments. The EOD width as an additional key parameter is not observable in the standard approaches. Therefore, qualitatively different EOD models (Fig. 5a vs. d), which potentially lead to different orientation optimization strategies, cannot be compared. Thus, the proposed method of emission lifetime analysis in modified OLED stacks exhibiting plasmonic Purcell effects could indicate a route to better understand the emitting material morphology, to tailor the emitter orientation and to significantly improve the device performance.

## Electronic supplementary material

Supplementary Information

## References

[1] Flämmich M (2011). Oriented phosphorescent emitters boost OLED efficiency. Org. Electron..

[2] Gather MC, Reineke S (2015). Recent advances in light outcoupling from white organic light-emitting diodes. J. Photon. Energy.

[3] Kim S-Y (2013). Organic light-emitting diodes with 30% external quantum efficiency based on a horizontally oriented emitter. Adv. Func. Mater..

[4] Graf A (2014). Correlating the transition dipole moment orientation of phosphorescent emitter molecules in OLEDs with basic material properties. J. Mater. Chem. C.

[5] Kim K-H (2014). Phosphorescent dye-based supramolecules for high-efficiency organic light-emitting diodes. Nature Commun..

[6] Jurow MJ (2016). Understanding and predicting the orientation of heteroleptic phosphors in organic light-emitting materials. Nature Mat..

[7] Mayr C (2014). Efficiency enhancement of organic light-emitting diodes incorporating a highly oriented thermally activated delayed fluorescence emitter. Adv. Func. Mater..

[8] Nakanotani H (2014). High-efficiency organic light-emitting diodes with fluorescent emitters. Nature Commun..

[9] Kaji H (2015). Purely organic electroluminescent material realizing 100% conversion from electricity to light. Nature Commun.

[10] Liehm P, Murawski C, Furno M, Lüssem B, Leo K (2012). Comparing the emissive dipole orientation of two similar phosphorescent green emitter molecules in highly efficient organic light-emitting diodes. Appl. Phys. Lett..

[11] Frischeisen J, Yokoyama D, Adachi C, Brütting W (2010). Determination of molecular dipole orientation in doped fluorescent organic thin films by photoluminescence measurements. Appl. Phys. Lett..

[12] Flämmich M, Michaelis D, Danz N (2011). Accessing OLED emitter properties by radiation pattern analyses. Org. Electron..

[13] Schmidt TD (2011). Evidence for non-isotropic emitter orientation in a red phosphorescent organic light emitting diode and its implications for determining the emitter’s radiative quantum efficiency. Appl. Phys. Lett..

[14] Penninck L, Steinbacher F, Krause R, Neyts K (2012). Determining emissive dipole orientation in organic light emitting devices by decay time measurements. Org. Electron..

[15] Irishina N, Moscoso M, Carminati R (2013). Recovering fluorophore location and orientation from lifetimes. Opt. Express.

[16] Schmidt TD (2014). Extracting the emitter orientation in organic light-emitting diodes from external quantum efficiency measurements. Appl. Phys. Lett..

[17] Purcell EM (1946). Spontaneous emission probabilities at radio frequencies. Phys. Rev..

[18] Penninck L, Mladenowski S, Neyts K (2010). The effects of planar metallic interfaces on the radiation of nearby emitters. J. Opt..

[19] Chance RR, Prock A, Silbey R (1978). Molecular fluorescence and energy transfer near interfaces. Adv. Chem. Phys..

[20] Danz N, Heber J, Kowarschik R, Bräuer A (2002). Fluorescence lifetimes of molecular dye ensembles near interfaces. Phys. Rev. A.

[21] Bartko AP, Xu K, Dickson RM (2002). Three-dimensional single molecule rotational diffusion in glassy state polymer films. Phys. Rev. Lett..

[22] Deschenes LA, Bout DAV (2001). Single-molecule studies of heterogeneous dynamics in polymer melts near the glass transition. Science.

[23] Lee M, Kim J, Tang J, Hochstrasser RM (2002). Fluorescence quenching and lifetime distributions of single molecules on glass surfaces. Chem. Phys. Lett..

[24] Drexhage KH (1974). Interaction of light with monomolecular dye layers. Prog. Optics.

[25] Steiner F, Bange S, Vogelsang J, Lupton JM (2015). Spontaneous fluctuations of transition dipole moment orientation in OLED triplet emitters. J. Phys. Chem. Lett..

[26] Chen W, Thoreson MD, Ishii S, Kildishev AV, Shalaev VM (2014). Ultra-thin ultra-smooth and low-loss silver films on a germanium wetting layer. Opt. Express.

[27] Flämmich M (2009). Dispersion-model-free determination of optical constants: application to materials for organic thin film devices. Appl. Opt..

[28] Palik, E. D. Handbook of Optical Constants of Solids, 353–359 (Academic Press, 1991).

[29] Gong J, Dai R, Wang Z, Zhang Z (2015). Thickness dispersion of surface plasmon of Ag nano-thin films: Determination by ellipsometry iterated with transmittance method. Sci. Reports.

[30] Wróbel P (2015). Ge wetting layer increases ohmic plasmon losses in Ag film due to segregation. Appl. Mater. Interf..

[31] Flämmich M, Danz N (2013). Optical characterisation of OLED emitters from radiation pattern analyses. Woodhead Publishing Series in Electronic and Optical Materials.

[32] Schmidt Tobias D., Setz Daniel S., Flämmich Michael, Scholz Bert J., Jaeger Arndt, Diez Carola, Michaelis Dirk, Danz Norbert, Brütting Wolfgang (2012). Degradation induced decrease of the radiative quantum efficiency in organic light-emitting diodes. Applied Physics Letters.

[33] Murawski, C., Elschner, C., Lenk, S., Reineke, S. & Gather, M. C. Orientation of OLED emitter molecules revealed by XRD. OSA Light, Energy and the Environment Congress, Leipzig, Germany, 14–17, paper SSW2D7 Nov, doi:10.1364/SSL.2016.SSW2D.7 (2016).

[34] Kawamura Y, Brooks J, Brown JJ, Sasabe H, Adachi C (2006). Intermolecular interaction and a concentration-quenching mechanism of phosphorescent Ir(III) complexes in a solid film. Phys. Rev. Lett..

[35] Weichsel C (2012). Storage of charge carriers on emitter molecules in organic light-emitting diodes. Phys. Rev. B.

[36] Pace MD, Barger WR, Snow AW (1989). Molecular Packing and Iodine Doping of Oxovanadium- and Copper-Substituted Tetrakis(cumy1phenoxy)phthalocyanine Langmuir-Blodgett Films Studied by ESR. Langmuir.

[37] Pace MD, Barger WR, Snow AW (1987). EPR of Copper Tetrakis(cumlphenoxy)phthalocyanine Langmuir-Blodgett Films. J. Magn. Res..

[38] Azumi R (1993). Orientation Change of Porphyrin in Langmuir-Blodgett Films Caused by a Trigger Molecule. J. Phys. Chem..

