# Supplementary material for: Plasmonic Purcell effect reveals obliquely ordered phosphorescent emitters in Organic LEDs
Source: Sci Rep. 2017 May 12;7:1826. doi: 10.1038/s41598-017-01701-8 (PMC5431857; doi:10.1038/s41598-017-01701-8)
Supplement: Supplementary file 1 — Supplementary Information [file 41598_2017_1701_MOESM1_ESM.pdf]

Supplementary Information

Plasmonic Purcell effect reveals obliquely ordered phosphorescent emitters in Organic LEDs

R. Mac Ciarnain, D. Michaelis, T. Wehlus, A. F. Rausch, S. Wehrmeister, T. D. Schmidt, W. Brütting, N. Danz, A. Bräuer and A. Tünnermann

PSTM layer dispersion and device reflectivity

The dispersion of the thin Germanium / Silver layer, introduced to cause orientation dependent losses for the emitters inside the EML, has been found not to agree with published values [1]. Dispersion data taken from Ref. 1 have been modified by adding a resonance in the red spectral region in order to estimate the real dispersion by a Cramers-Kronig consistent modification. Supplementary Figure S1 plots the original and modified data for convenience. Note that such dispersion compares well to previous results [2].

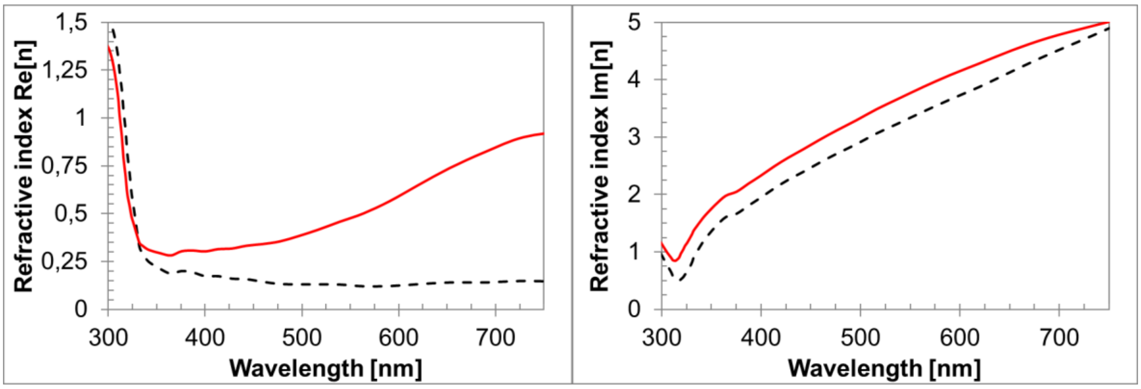

Supplementary Figure S1: Dispersion of the refractive index real (left) and imaginary (right) parts versus wavelength. Tabulated data taken from Ref. [1] is plotted dashed black while the modified dispersion is shown straight red.

The application of this dispersion is illustrated in the Supplementary Figure S2 that plots the unpolarized reflectivity of the full OLED stack that has been measured at an angle of 6%. The simulations are plotted without further fitting of the layer thicknesses and agree very well with the experimental data in the 500…700 nm spectral range of OLED emission.

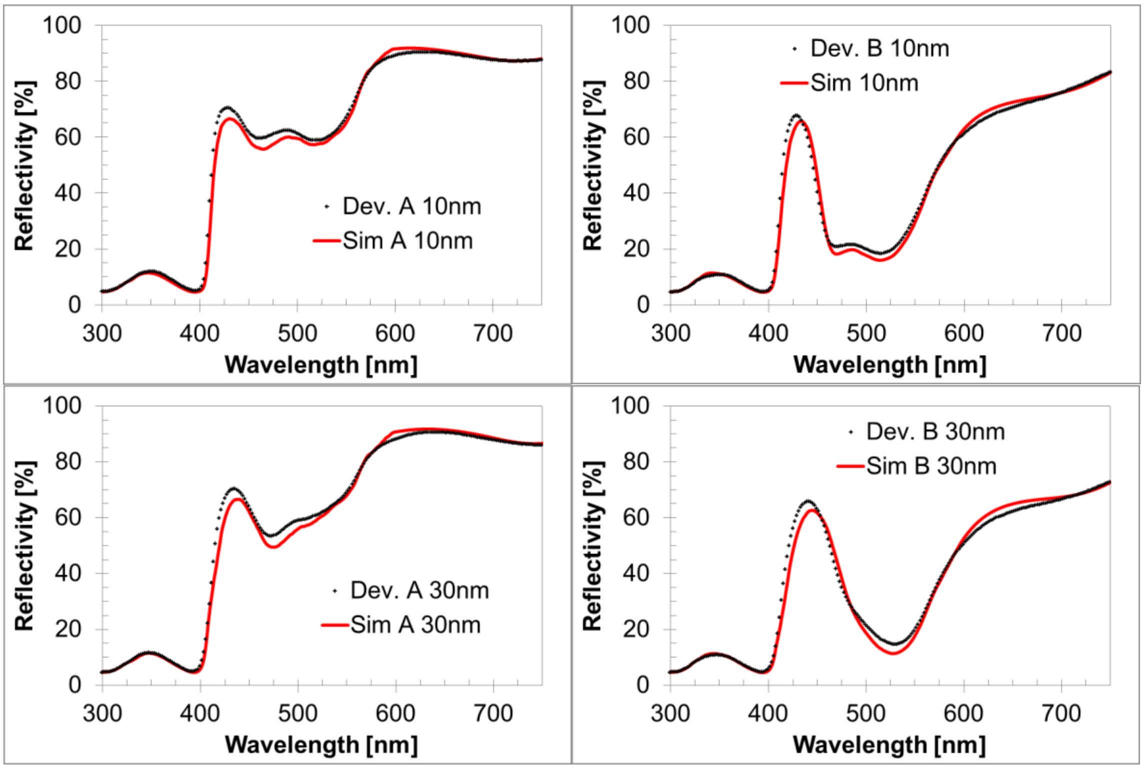

Supplementary Figure S2: Reflectivity data (black crosses) measured at 6° angle of incidence at the complete OLED stacks are plotted along with the simulation (red curve) when assuming the indicated thicknesses of the HTL spacer layer.

Emitter orientation analysis

The average emitter orientation was derived from emission pattern measurements as illustrated in Figure 4 of the paper. Unfortunately, such plots do not reveal deviations between experimental and simulated data well. Therefore, the Supplementary Figure S3 plots the TM polarized emitted intensity at 650 nm wavelength along with a simulation for four different devices. Such wavelength illustrates the presence of perpendicular emitter components very well as it reveals the maximum separation of emitted intensities originating from parallel and perpendicular components of the ensemble’s transition dipole moments.

For devices B the data noise increases due to the low level of light emitted into air. However, the model plotted in Supplementary Figure S3 assuming $\langle p_{\parallel}^2 \rangle_{\varphi} : \langle p_{\perp}^2 \rangle_{\varphi} = 2:0.69$ fits the experimental data well. Therefore, we conclude no effect of the presence of the PSTM layer on the average orientation of the emitting ensemble.

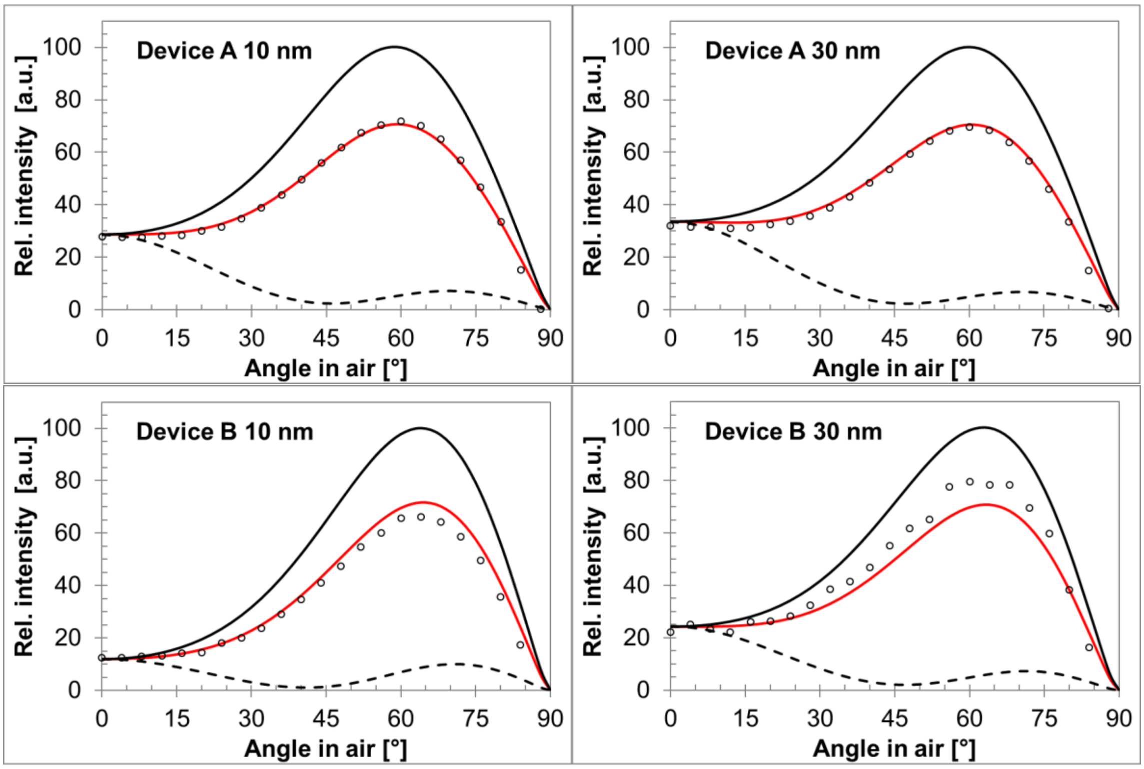

Supplementary Figure S3: Relative intensity vs. observation angle in air obtained for TM polarized emission at (650±2) nm wavelength. The diagrams include experimental data (circles) as well as simulated intensities when assuming ensemble orientation averages $\langle p_\parallel^2 \rangle_\varphi : \langle p_\perp^2 \rangle_\varphi$ *of 2:1 (isotropic, straight black), 2:0.69 (aligned, straight red), and 2:0 (strictly parallel, dashed black). Data is normalized to the maximum of the isotropic ensemble simulation in order to ease direct comparison.*

PL emission lifetime analysis

The transient luminescence of the samples has been recorded following short pulse optical excitation. The temporal luminescence decays are shown in Supplementary Figure S4. The data recorded by the oscilloscope have been temporally shifted in order to assign the time t=0 to the onset of the PL decay when laser excitation is turned off. All traces were then fitted using the formula

$$I(t) = (I_0 - I_{BG}) \cdot \exp(-t/\tau) + I_{BG}$$

with the intensity amplitude I_0 at onset of decay, the background intensity I_{BG} without excitation, and the emission lifetime τ. The results in Supplementary Figure S4 below illustrate the quality of fitting and the differences due to introduction of the PSTM layer into the device. Furthermore, the observation polarization (difference between red and blue colored data) has negligible effect on the emission time trace, thus indicating the presence of a narrow, aligned ensemble.

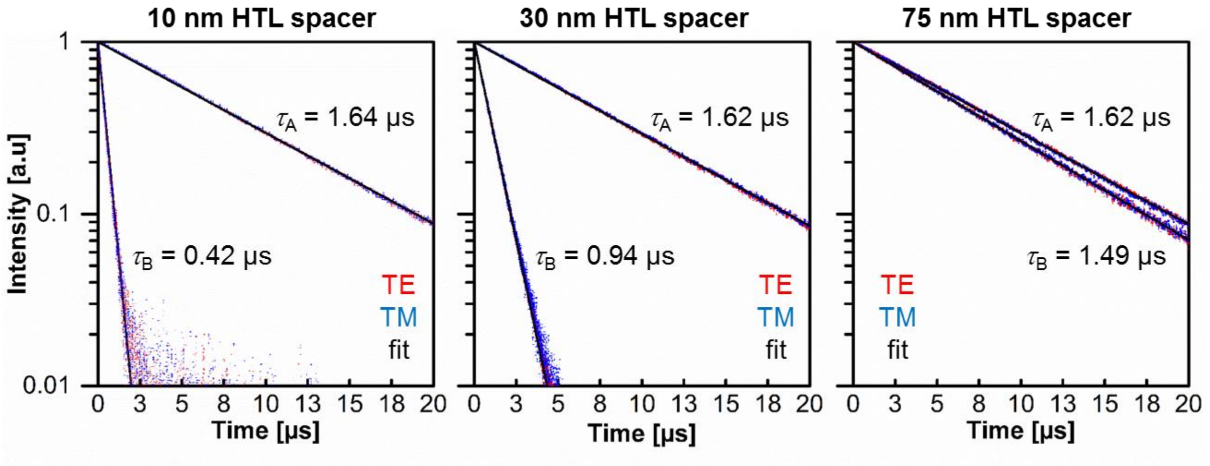

Supplementary Figure S4: Transient PL data plotted for device types A and B with three different HTL spacer thicknesses. The diagrams show experimental data for TE (red) and TM (blue) polarized observation along with the corresponding single exponential fit results and the lifetimes derived. Measurement error of emission lifetime is estimated to be in the $\Delta\tau=\pm0.02$ µs range. Amplitudes have been normalized for convenience.

References

[1] Palik, E. D., Handbook of Optical Constants of Solids, Academic Press, 1991.

[2] J. Gong, R. Dai, Z. Wang, and Z. Zhang, Thickness Dispersion of Surface Plasmon of Ag Nano-thin Films: Determination by Ellipsometry Iterated with Transmittance Method, Sci. Reports **5**, 9279 (2015).
